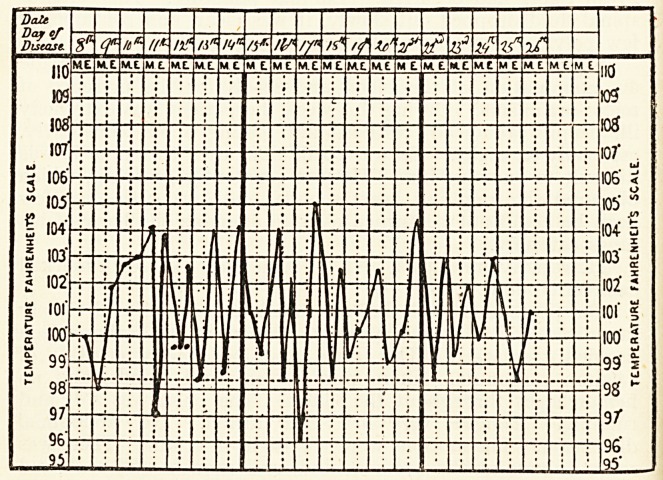# A Case of Acute Septicæmia Due to the B. Pyocyaneus

**Published:** 1913-03

**Authors:** J. Michell Clarke

**Affiliations:** Professor of Medicine, University of Bristol; Physician to Bristol General Hospital.


### A CASE OF ACUTE SEPTICÆMIA DUE TO THE B. PYOCYANEUS.

BY

J. Michell Clarke, M.A., M.D., LL.D., F.R.C.P.,
*Professor of Medicine, University of Bristol; Physician to Bristol General Hospital.*

Cases of "cryptogenetic" septicæmia are always of interest, and the B. pyocyaneus is not one of the common organisms in septicæmia, although it has been found more frequently with the improvement of bacteriological methods. Muir and Ritchie state that the B. pyocyaneus is generally found in association with other micro-organisms, but that it has been found lately in some diseases in children as a general infection in pure culture, the chief symptoms being fever, gastro-intestinal disturbances, hemorrhages, petechial or pustular eruptions, and general marasmus. More recently it has been found in many cases in adults as the sole cause of a general infection (septicæmia).

Waite, in a paper[1] in which a full bibliography and account

[1] "Contribution to Study of Pyocyaneous Infections," *J. Infect. Dis.*, 1908, v. 542.

of published cases to 1908 is given, states that the B. pyocyaneus has been recovered in pure culture from all parts of the body, in local lesions and general systemic infections, and from the blood during life in not a few instances. He states that certain parts of the body are especially apt to be affected, especially the middle ear and the alimentary tract, and that it may be the specific cause of both sporadic and epidemic diarrhœa, and of dysentery. There are several outbreaks of diarrhœa in infants or children recorded as due to this organism, and it may also give rise to acute appendicitis (Kelly).

In an epidemic of dysentery reported by Lartigau,[1] fifteen persons were affected, of whom four died. The B. pyocyaneus was found in the stools, and also in the water of the wells used by the patients, so that in this outbreak the mode of infection seems clear. He points out an important fact in diagnosis, namely that the organism may not produce blue pigment for some days, and perhaps not at all until it has been passed through animals.

The B. pyocyaneus is found occasionally in peritonitis of various origin, in pleurisy, infective endocarditis, in meningitis, in liver abscesses and in cystitis, either alone or with other organisms. It is also sometimes present in cutaneous lesions, where these are either only a local manifestation, or part of a more general infection. In pemphigus, either true or false, it seems especially prone to occur.[2]

Ernst thinks that there are two varieties of B. pyocyaneus, the one producing the chronic the other the acute lesions.

From the published cases children seem to be more susceptible to infection than adults. I pass over the well-known infection of wounds, etc., by this organism. With regard to B. pyocyaneus as a cause of general septicæmia, Waite thinks that the infection may arise from any primary focus, but most frequently from the intestinal tract, and quotes Lannay as to the frequent origin of general pyocyaneus infections from

[1] Quoted by Waite, *loc. cit.*, and by Roberts, *Tr. Coll. Phys. Phil.*, 1905, 3rd Ser., xxvii. 137.

[2] Petges and Bichelonne, *Ann. de Dermat. et de Syph.*, 1908, ix. 417.

primary intestinal lesions, and as to the recovery in such cases of the bacillus from the blood, fæces, mesenteric glands, and ecchymoses of intestinal mucosa.

Charrin and Ehlers each report two cases, in each instance occurring in a brother and sister, of general pyocyaneus septicæmia. In each case the symptoms resembled those of either enteric fever or cerebro-spinal meningitis ; one patient died of enteritis ; there were vesicular or papular eruptions, which in one case went on to the formation of pustules having a bluish colour.

The account of my case is as follows :—

The patient was a labourer, aged 15. His mother died of pulmonary tuberculosis, and one brother of peritonitis. No previous illness. He had had a slight cough, mostly in the mornings, for several months. No discharge from ears or nose. His work consisted of turning on steam for a steam winch.

On September 7th, 1912, he was suddenly taken with headache, drowsiness and vomiting ; there were no rigors. He felt ill, but kept about until September 10th, when he took to his bed, where he remained, and complained chiefly of headache, drowsiness, weakness, and slight cough. There was no return of the initial vomiting, no diarrhœa, no jaundice, no photophobia, no pains in limbs. On admission September 12th patient was weak and drowsy, he lay on his back with his eyes closed, and complained of frontal headache. Pulse 82, regular. Temperature 100°. Respiration 20. Bowels constipated. Tongue furred thickly. Chest : except for the signs of bronchitis, the lungs were normal.

The cardiac apex-beat, area of dulness and sounds were normal. Abdomen slightly retracted and hollow.

Liver dulness normal, edge not felt below ribs. Spleen not enlarged. Urine acid, no albumin or sugar.

The nervous symptoms predominated at this time. He was very drowsy, but sensible ; answered when spoken to, and was not delirious.

All reflexes, superficial and deep, were present, and equal on both sides. The cranial nerves were not affected. Pupils normal, acted well. Optic discs normal.

September 14th.—Lumbar puncture showed cerebro-spinal fluid to be under moderate pressure. It was clear and contained a large number of lymphocytes, some epithelioid cells, but no organisms.

September 17th.—He had a distinct rigor. For one or two days after lumbar puncture he had increased headache.

September 19th.—More drowsy. He goes off to sleep directly after feeding; there is photophobia, and he covers his head with the bedclothes, otherwise no change in his condition, and no fresh physical signs of organic disease in nervous system or lungs. The bronchitis continued. Profuse sweats. The case at this stage resembled, clinically, one of meningitis.

September 23rd.—There was distinct jaundice, edge of liver was one inch below the costal margin, splenic dulness was increased, and the tip felt. A systolic murmur appeared over the base of the heart. No hemorrhages or petechiæ into skin. Fresh rigors had occurred. Lumbar puncture. The cerebro-spinal fluid dropped out slowly, it was clear, and contained a few polymorphonuclear cells and no organisms.

September 25th.—On this day Mr. Scott-Williamson made an examination of the blood and stools for the presence of micro-organisms. During this week the patient was extremely ill, deeply jaundiced, restless, and tossing about constantly, especially at night. He was so ill that after this time it was difficult to examine him. He was constantly groaning, which obscured the lung and heart sounds, and during the last few days he could not be turned over to examine the back of his lungs carefully.

He rapidly emaciated, and for some days exhaled a marked cadaveric odour.

On September 30th he was much emaciated and deeply jaundiced. The liver appeared reduced in size, the spleen decidedly enlarged. The urine contained many crystals of calcium carbonate. There were no enlarged glands, and no hemorrhages throughout the illness. The pulse was soft and running. Tongue very dry and furred. No abdominal distension. The motions were very light in colour. There was some dulness at the bases behind, and rhonchi and scattered moist crepitations over both lungs, but his constant groaning interfered with auscultation.

*Blood.* — Erythrocytes, 2,890,000; leucocytes, 14,000; hæmoglobin, 35 per cent.

Differential count—

| | | |
|---|---|---|
| Polymorphs | .. .. .. | 51.2 per cent. |
| Transitionals | .. .. .. | 1.7 ,, |
| Small lymphocytes | .. .. | 16.5 ,, |
| Large ,, | .. .. | 7.2 ,, |
| ,, mononuclears | .. .. | 21.5 ,, |
| Eosinophils | .. .. .. | 1.2 ,, |
| Basophils | .. .. .. | .2 ,, |

1 normoblast in 400 whites counted.

*Urine* contained urea, 2 per cent.; a large amount of indican; no leucin or tyrosin; large numbers of acicular and stellar forms of crystals of calcium phosphates and of crystals of calcium carbonate ; albumin and bile. Fæces showed presence of bile pigment, traces of blood, pus cells, and much mucus.

On October 1st Mr. Scott-Williamson reported that he had recovered the B. pyocyaneus in pure culture from the blood and fæces, and had prepared vaccines. The bacillus agglutinated 1–40 of its own organism, and 1–80 of a stock culture.

He died early on October 2nd, before the vaccines could be utilised.

The *post-mortem* examination was made by Mr. Scott-Williamson. There were pleural and pericardial adhesions. The right pleura contained about twenty ounces of thick greenish-blue, odourless fluid. The left was empty.

*Right Lung.*—A large abscess communicating with the pleura was found in the posterior part of the lower lobe. There were many small abscesses scattered through this lobe. The abscesses apparently had not originated in connection with the bronchi ; each had a distinct wall, and contained greenish-blue pus.

*Left Lung.*—Contained many small abscesses, especially in its lower lobe.

In both lungs were scattered small areas of broncho-pneumonic consolidation.

The glands at the root of the lungs were swollen and soft.

*Heart.*—Slight fatty change in muscular wall (microscopically).

*Abdomen.*—Peritoneum reddened; its cavity contained no fluid or pus.

Recent adhesions united the upper surface of the liver to the diaphragm.

*Liver.*—Large and congested, and microscopically showed slight fatty degeneration.

*Spleen.*—Large and very soft, almost diffluent. The Malpighian bodies stood out prominently. Microscopically there was an excess of polymorphonuclear cells, and active proliferation of lymph nodes.

*Kidneys.*—Congested, the glomeruli especially so.

*Nervous System.*—The cerebral meninges were hyperæmic, but not thickened, and there was no purulent exudate. The brain-cortex was injected and somewhat soft.

The B. pyocyaneus was recovered in pure culture from the heart's blood, pleural exudate, and lung abscesses, but not from the spleen or brain.

In this patient, as in the cases of general pyocyaneous infection referred to above, the nervous symptoms predominated during the first part of the illness, recalling the resemblance to cerebro-spinal meningitis remarked upon by other writers. These symptoms were amply explained at the *post-mortem* by the hyperæmia of the meninges and brain-cortex. The lymphocytosis of the cerebro-spinal fluid removed by spinal puncture pointed to meningeal irritation, but the absence of any micro-organisms in the fluid was against an infective cerebro-spinal meningitis. I do not attach great importance in differential diagnosis to severe nervous symptoms in such cases, as they so often dominate the clinical aspect in any acute septicæmia in its early stages. Thus nervous symptoms predominated in two other cases of general infection of unusual origin recently under my care, the one due to a pathogenic leptothrix, and the other to the B. influenzæ. I think that the *post-mortem* appearances do not altogether clear up the source of infection in this case. It may have been from the lungs, in which the chief lesions were found, or from the intestinal tract, as suggested by the occurrence of jaundice. Clinically, signs of bronchitis appeared some days before jaundice, and so far as this goes it points to a primary lung infection. The more severe lesions of the lung must have occurred during the last week of the patient's life, and were masked by the severity of the general illness.

In conclusion, it is possible that the relative increase of the large mononuclear cells of the blood, in the presence of a slight leucocytosis, is a feature of diagnostic importance. The very peculiar cadaveric odour exhaled from the patient may also be mentioned.

I am much indebted to Mr. Scott-Williamson for the thorough way in which the bacteriological investigation of the disease was worked out.

## Figures and Tables

**Figure f1:**